# Assessing and Mapping Spatial Associations among Oral Cancer Mortality Rates, Concentrations of Heavy Metals in Soil, and Land Use Types Based on Multiple Scale Data

**DOI:** 10.3390/ijerph110202148

**Published:** 2014-02-21

**Authors:** Wei-Chih Lin, Yu-Pin Lin, Yung-Chieh Wang, Tsun-Kuo Chang, Li-Chi Chiang

**Affiliations:** 1 Department of Bioenvironmental Systems Engineering, National Taiwan University, No. 1, Section 4, Roosevelt Road, Da-an District, Taipei City 106, Taiwan; E-Mails: B97602046@ntu.edu.tw (W.-C.L.); beckyycwang@gmail.com (Y.-C.W.); tknchang@ntu.edu.tw (T.-K.C.); 2 Department of Civil and Disaster Prevention Engineering, National United University, 2 Lien-Da, Nan-Shih-Li, Miaoli 36003, Taiwan; E-Mail: lchiang@nuu.edu.tw

**Keywords:** spatial deconvolution, spatial correlation, oral cancer, heavy metals, land use, p-field simulation, kriging

## Abstract

In this study, a deconvolution procedure was used to create a variogram of oral cancer (OC) rates. Based on the variogram, area-to-point (ATP) Poisson kriging and p-field simulation were used to downscale and simulate, respectively, the OC rate data for Taiwan from the district scale to a 1 km × 1 km grid scale. Local cluster analysis (LCA) of OC mortality rates was then performed to identify OC mortality rate hot spots based on the downscaled and the p-field-simulated OC mortality maps. The relationship between OC mortality and land use was studied by overlapping the maps of the downscaled OC mortality, the LCA results, and the land uses. One thousand simulations were performed to quantify local and spatial uncertainties in the LCA to identify OC mortality hot spots. The scatter plots and Spearman’s rank correlation yielded the relationship between OC mortality and concentrations of the seven metals in the 1 km cell grid. The correlation analysis results for the 1 km scale revealed a weak correlation between OC mortality rate and concentrations of the seven studied heavy metals in soil. Accordingly, the heavy metal concentrations in soil are not major determinants of OC mortality rates at the 1 km scale at which soils were sampled. The LCA statistical results for local indicator of spatial association (LISA) revealed that the sites with high probability of high-high (high value surrounded by high values) OC mortality at the 1 km grid scale were clustered in southern, eastern, and mid-western Taiwan. The number of such sites was also significantly higher on agricultural land and in urban regions than on land with other uses. The proposed approach can be used to downscale and evaluate uncertainty in mortality data from a coarse scale to a fine scale at which useful additional information can be obtained for assessing and managing land use and risk.

## 1. Introduction

The occurrence of oral cancer (OC) has increased rapidly in Taiwan during the last three decades. The incidence of OC in males increased 5.3-fold from 1982 to 2001 [1]. Oral cancer is the fourth most common cause of cancer-related deaths in Taiwan and the most common cause of cancer-related deaths in males [2,3]. Heavy metals concentrations in soil and vegetation are known to be directly or indirectly related to cancer occurrence [1,2,3,4]. Some studies [1,2,3] have indicated that, since OC is spatially related to chronic exposure to heavy metals, including arsenic (As), chromium (Cr), and nickel (Ni), the distributions of these heavy metals can be used to map the distribution of cancer occurrence in Taiwan. Moreover, based on the results of the spatial lag model, Chiang *et al.* [3,4] have shown that, in Taiwan, the concentrations of Ni and Cr in soil are spatially correlated with OC mortality. 

Su *et al.* [2] has shown that the prevalence of betel quid chewing and cigarette smoking is not highly related to the high OC mortality in Changhua County in Taiwan, which is, in fact, caused by the pollutants in land with various uses, such as by the heavy metals released by electroplating factories. The high mortality rates associated with nasopharyngeal, lung, intestinal, and mesothelioma cancers have shown significant associations with two land use types agricultural and urban [5]. Relevant studies have indicated that heavy metal enrichment in soil is associated with human activities, especially in built-up areas and on agricultural land [6,7,8,9,10]. Su *et al.* [1] suggested that, along with betel quid chewing and cigarette smoking, long-term exposure to As or Ni is a risk factor for OC. Su *et al.* [1] also confirmed the finding by Yuan * et al.* [11] that oral cancer is significantly related to levels of Ni and Cr in the blood, which can be affected by long-term exposure to Ni and Cr in the environment. However, cancer mortality data are usually aggregated over irregular spatial supports (scales) (such as counties or districts) and consist of a numerator and a denominator (rate) [12]. Oral cancer mortality rates, the data of soil samples, and land uses are usually recorded on various scales, causing potential difficulties in evaluating the strengths of their relationships. Therefore, this study disaggregated these data and mapped them at a scale at which the relationships among OC mortality rate, soil samples data, and land use could be identified effectively. 

In epidemiology, area-to-point kriging is increasingly used to change the support for incidences of cancer and other diseases and to map cancer mortality distributions. Area-to-point (ATP) Poisson kriging can be used to obtain maps of mortality risk in which the visual bias associated with map interpretation is reduced [13]. Since ATP Poisson kriging can effectively downscale and map cancer rates [13,14,15,16,17], cancer rate maps generated by this kriging method can reveal potential “hot spots” that are not evident when aerial or aggregated data are used [14]. ATP kriging has also been used to estimate the distribution of cancer mortality rates [13,16,17]. Recently, Goovaerts [18] used ATP binomial kriging to map prostate cancer rates by combining individual-level data and census tract-level data. Bonyah *et al.* [19] used Poisson kriging to filter the noise in Buruliulcer incidence data, map the corresponding risk at a finer resolution, and estimate geographical clustering of the disease at the scale of administrative units. Local cluster analysis can be used to detect hot spots of OC mortality rate using a downscaled OC mortality map. Although ATP Poisson kriging provides the kriging variance that is required to measure the uncertainty, it cannot be used directly to quantify the uncertainty of statistics such as the Local Indicator of Spatial Autocorrelation (LISA) statistic [20]. However, combining Poisson kriging with stochastic simulation can generate multiple realizations of the spatial distribution of disease risk to quantify uncertainty in the spatial distribution of health outcomes, which can then be translated into uncertainty in the locations of disease clusters [21,22], the presence of significant boundaries [23], or the relationship between health outcomes and putative risk factors [20]. Accordingly, multi-location uncertainty, which is the jointly prevailing uncertainty at many specific locations, can be used to evaluate the reliability of delineation hot spots based on the probability of the hotspot area [20].

Local Cluster Analysis (LCA) using the LISA statistic is widely used to identify local clusters or outliers of high or low cancer risk [12,20,24,25]. The LISA value, and hence the conclusion about the presence of clusters and outliers, is related to the neighborhood structure [20]. A negative LISA statistic indicates a negative local auto-correlation and the presence of a spatial outlier whose kernel value is much lower (higher) than the mean of the surrounding values [20]. Clustering of low (high) values results in positive values of the LISA statistic. Any studied unit (county, district, or grid node or raster cell) with a p-value lower than the significance level is classified as a significant spatial outlier (HL: high value surrounded by low values, and LH: low value surrounded by high values), or a cluster (HH: high value surrounded by high values, and LL: low value surrounded by low values) [20]. Other studies [12,20] have also successfully used ATP kriging and LCA to identify clusters of low (LL) and high (HH) cancer mortality risk in various areas. Relationships between cancer mortality risk and land use can be identified by overlapping the downscaled and the simulated OC mortality LISA and land use maps.

This study calculated variogram of OC rate by using a deconvolution procedure. Based on the de-convoluted variogram of the OC rates, ATP Poisson kriging was used to downscale the OC rate data for Taiwan from the district-level scale to a 1 km × 1 km grid scale at which soil and land use samples were obtained. The scatter plots and Spearman's rank correlation yielded the relationship between the OC mortality and the concentrations of the seven metals in soil in the grid of 1 km cells. LCA was used to obtain LISA statistics to identify hot spots of OC mortality rate based on the downscaled OC mortality map. Moreover, the LISA values on each map which was generated by the p-field simulation were computed to illustrate how the uncertainty of the HH (hotspot) affected theOC mortality rate in the delineated hotspots. The relationship between OC mortality and land use was identified by overlapping the maps of the downscaled and the simulated OC mortality, the LISA statistic, and land use.

## 2. Experimental Section

### 2.1. Data Collection

Data on oral cancer mortality rates were obtained from the Atlas of Cancer Mortality in Taiwan [3]. The data included the OC age-standardized mortality rates (ASMR) of males in 343 districts from 1972 to 2001 [3]. Heavy metals concentrations were obtained from a nationwide survey performed by the Environmental Protection Administration (EPA) in Taiwan and included concentrations of arsenic (As), cadmium (Cd), chromium (Cr), copper (Cu), nickel (Ni), lead (Pb), and zinc (Zn) in soil at depths of 0–15 cm. Samples were obtained using a grid of 100 ha cells. The heavy metal concentration in 100 ha was the average of that in 30 points. A total of 2,183 soil samples were collected across Taiwan, except in the center of the study area. The data of the heavy metal concentrations in soil were obtained from Chiang *et al.* and Su *et al.* [1,3]. The scatter plots between oral cancer mortality rate and heavy metal concentrations in soil were performed to confirm their spatial correlation.

**Figure 1:**
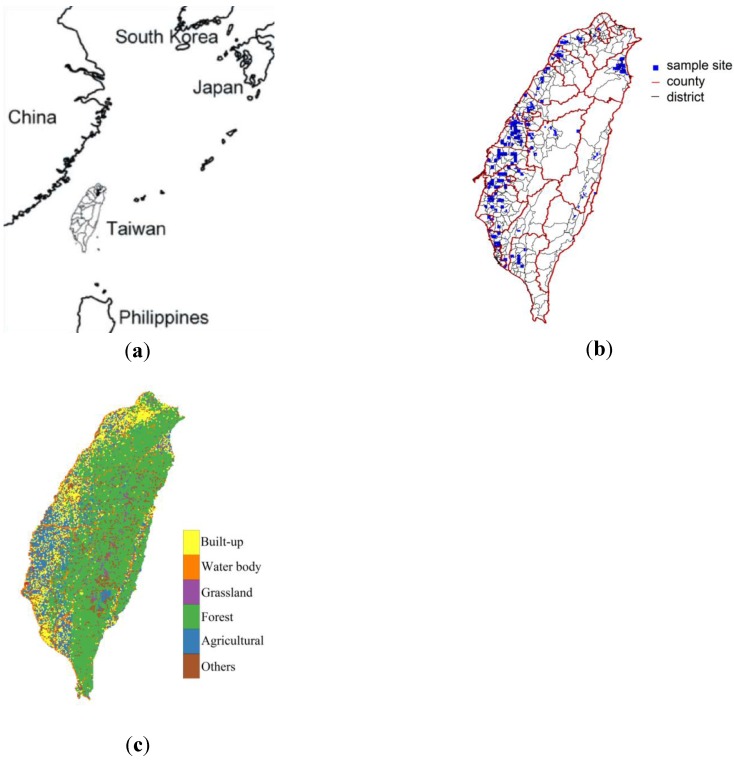
(**a**) Location of study area. (**b**) Locations of heavy-metal samples. (**c**) Land uses in study area*.*

Land use data for year 1999 were obtained from the Soil and Water Conservation Bureau of the Council of Agriculture, Taiwan. Land use maps generated and digitized by the Soil and Water Conservation Bureau based on 1:5,000 aerial photographs taken in 1999 were used to distinguish among 33 land uses in a vector format. Based on the definitions of land uses that are used by the Construction and Planning Agency of the Ministry of the Interior, Taiwan, land uses were reclassified into five broad classes—agricultural, forest, built-up, grassland, and water body (Figure 1c).

### 2.2. Area-to-point Poisson Kriging

Before performing ATP kriging, the point support covariance of risk *C_R_*(*h*) or the semivariogram *γ_R_*(*h*) was obtained, and an iterative de-convolution procedure was used to calculate the area-to-area and area-to-point covariance of the risk. The de-convolution procedure and estimator of OC mortality variogram detailed by Goovaerts (2006) [15] was used to de-convolute the semivariogram of the risk. This procedure was performed by an R program. Kriging, a best linear unbiased estimator, is a method of interpolation in which the interpolated values are modeled using a Gaussian process that is governed by a spatial correlation. Moreover, kriging depends on the expression of a spatial correlation, and it minimizes the prediction errors, which are themselves estimated based on observed values of the variable of interest [26]. A semivariogram was used herein to represent spatial correlation, and is defined using three parameters, which are the nugget effect, the sill and the range. The nugget effect represents the micro-scale variation or measurement error. The sill represents the variance of the random field. The range is the distance at which data are no longer autocorrelated.

The formula used to calculate the observed mortality rate in area , *v_i_*, *z*(*v_i_*) [13,20] was:

*z*(*v_i_*) = *d*(*v_i_*)/ *n*(*v_i_*) (1)

where *n*(*v_i_*) is the population in area *v_i_*; *d*(*v_i_*) is the number of cases of OC mortality, and *D*(*v_i_*) is a random variable that follows a Poisson distribution with an expectation of *n*(*v_i_*) × *r*(*v_i_*), where *r*(*v_i_*) is the local risk of OC mortality. 

The OC risk at location *u_α_*, *r*(*u_α_*) is estimated as a linear combination of *K* neighboring risks [13,20]:

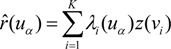
(2)
where *λ_i_*(*u_α_*), which is the kriging weight at location *u_α_*, is obtained by solving the following system of equations:

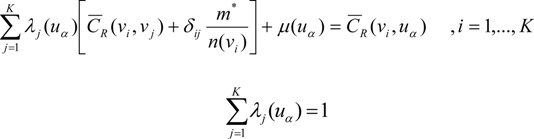
(3)
where *δ_ij_* = 1 if *i = j* and *δ_ij_* = 0 otherwise; m* is the population-weighted mean of the N rates, and *μ*(*u_α_*) is the Lagrange multiplier. Area-to-area covariance *C_R_* (*v_i_*, *v_j_*) is *Cov*{*Z*(*v_i_*), *Z*(*v_j_*)}, which is the weighted average of the point-support covariance between any two locations in areas *v_i_* and *v_j_*. Area-to-point covariance *C_R_* (*v_i_*, *u_α_*) is the weighted average of the point-support covariance between any location in area *v_i_* and location *u_α_*:

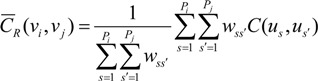
(4)

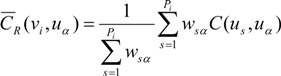
(5)
where *P_i_* and *P_j_* are the numbers of points used to discretize the two areas *v_i_* and *v_j_*, respectively. Weight *w_ss'_* is the product of population sizes within points *n*(*u_s_*) and *n*(*u_s'_*):

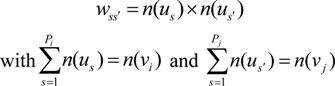
(6)


The kriging variance associated with the ATP Poisson kriging estimator is:

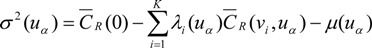
(7)


### 2.3. Cluster Analysis

The similarity between OC mortality rate at site *v*_*α*_and the average rate recorded over *j*(*v_α_*) neighboring geographical units *v_α_* is measured by [14,22,27] :

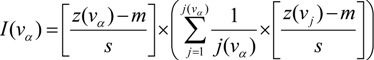
(8)
where *m* and *s* are the mean and standard deviation, respectively, of the set of mortality rates at N sites; *z*(*v_α_*) is the OC mortality rate at site *v_α_*; similarly, *z*(*v_i_*) is the OC mortality rate at site *v_i_*, and *j*(*v_α_*) is the number of neighboring sites *v_α_*.

This LISA value is the product of the kernel rate and the average of the neighboring kernel rates. The empirically determined LISA values computed using the above equation are compared with the value implied by the null hypothesis of complete spatial randomness. However, ATP Poisson kriging provides the kriging variance, which cannot directly quantify the uncertainty of the LISA statistic. As described by Goovaerts and Kerry *et al.* [21,22], this uncertainty was quantified by analyzing 1,000 p-field simulated OC mortality rate maps. The LISA values computed for each map generated by the p-field simulation [21] were used to evaluate how the uncertainty in OC mortality rates impacted the delineation of hotspot areas. Correlations of LISA values between two areas were tested in 1,000 simulations in order to compute the probability of an area belonging to a local cluster and the probability of an area being a spatial outlier [21]. Based on the p-field simulation, the *l*th realization of the OC mortality rate at site *u_α_*, *r*^(*l*)^(*u_α_*) was computed using the following equation:

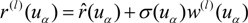
(9)
where *l* = 1,...,1000; {*w*^(*l*)^(*u_α_*), *α* = 1,...,*B*} are generated using non-conditional sequential Gaussian simulation and using the semivariogram of the risk, rescaled to a unit sill [21,22], and B is the total number of 1 km × 1 km cells. Further details and simulation results for this procedure can be found in the literature [21,22,23].

This study used stochastic simulations to map OC mortality rates simultaneously at many locations within the study area. The critical proportions, which are the number of realizations where the location is classified as HH clusters (joint probabilities), were 95%, 96%, 97%, 98%, and 99%. These proportions were used to measure spatial uncertainties when delineating the HH areas of OC mortality. The joint probability (P*_j_*, spatial uncertainty) associated with HH OC mortality rate in m HH locations (*u*_1_, *u*_2_, *u*_3_, …, *u_m_*) is written as follows [20]:

P*_j_* = *n_p_* (*u*_1_, *u*_2_, *u*_3_, …, *u_m_*)/ 1000 (10)

where 1,000 is the number of simulations, and *n_p_* (*u*_1_, *u*_2_, *u*_3_, …, *u_m_*) is the number of the 1,000 realizations in which all simulated OC mortality rates at m locations are HH; m is the number of the locations in which the HH covered proportions in the 1,000 p-field realizations exceeds the critical proportions; n_p_ is the number of realizations in which the simulated OC mortality rates at those m locations are all HH in each realization in the 1,000 realizations.

### 2.4. Kruskal-Wallis Test for Comparing Mean Mortality Rates for All Land Use Types

In the analysis of variance performed to determine whether the mean mortality rate for each land use type significantly differed from that throughout the study area, a Box-Cox transformation was attempted to convert the distributions of these rates into normal distributions. However, this method failed. Therefore, a nonparametric Kruskal-Wallis test was performed to determine whether the median mortality rates for the six land use types significantly differed from each other. Before the Kruskal-Wallis test, the Levene test was performed to test the homogeneity of the variance in OC mortality rate across the six land use types. When a significant difference was identified by Kruskal-Wallis test, a one-tailed multiple comparison test was then performed to identify significant differences in the mortality rates among the six land use types.

## 3. Results

### 3.1. Downscaled Spatial Distribution and Variance in OC Mortality Rate

**Figure 2:**
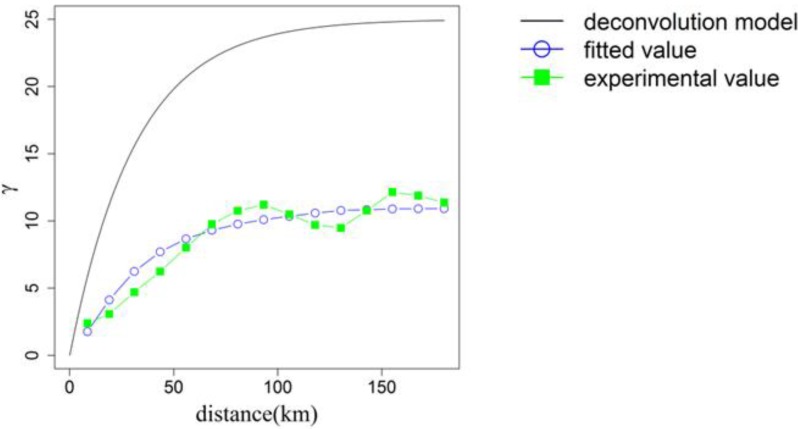
Experimental variograms of OC mortality of males (per 100,000 people)^2^, and experimental variograms fitted by exponential model and de-convolution models over Taiwan island.

**Figure 3:**
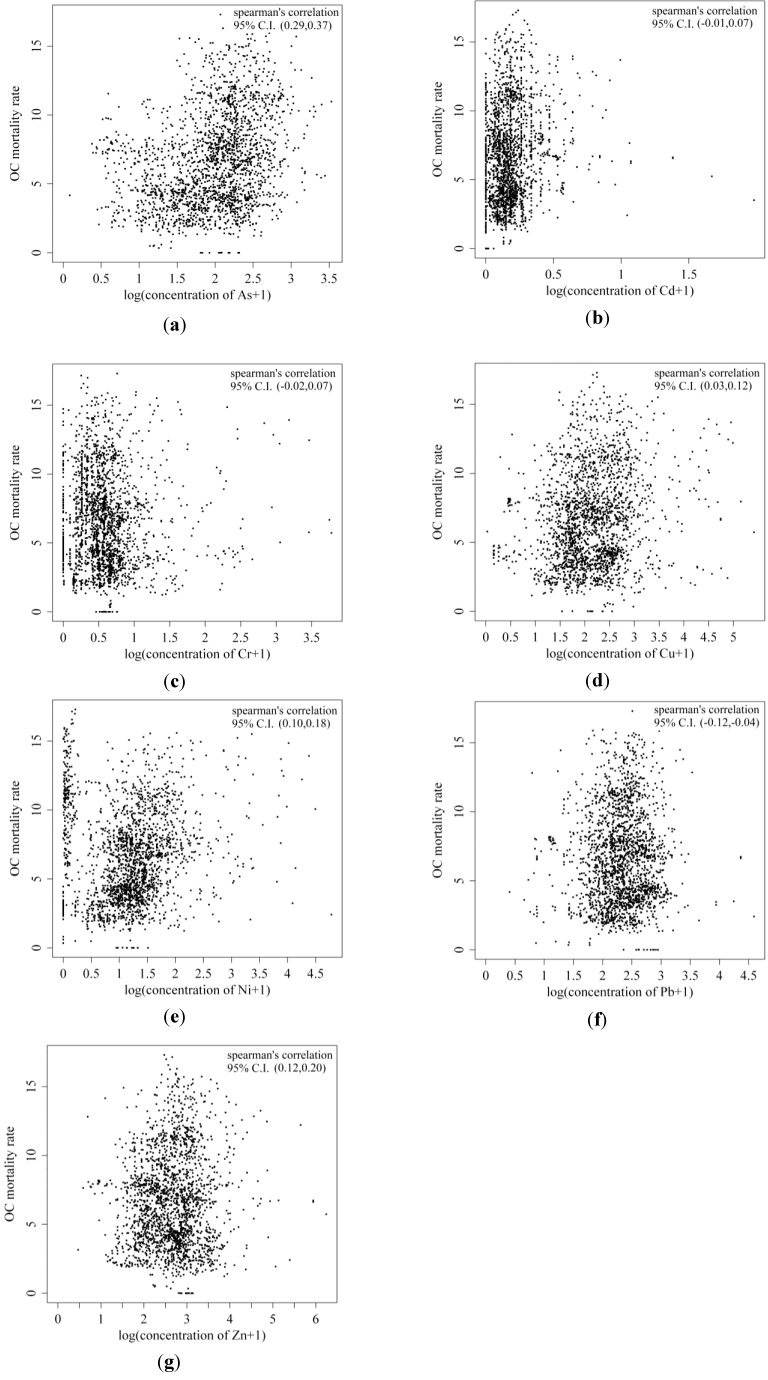
The scatter plots and the 95% confidence intervals of Spearman’s rank correlation between (**a**) As and OC, (**b**) Cd and OC, (**c**) Cr and OC, (**d**) Cu and OC, (**e**) Ni and OC, (**f**) Pb and OC, (**g**) Zn and OC.

Figure 2 shows the omni-directional variogram of the oral cancer mortality rate of males at the district scale. The presented de-convolution models were used to convert the support data from the district scale to the 1km scale. The experimental variogram was de-convoluted using the point support model (exponential model) using an iterative algorithm. De-convolution models with a range of 90 km were used to estimate the mortality rates in 1 km cells over the entire study area. The variograms on the district-level scale and the 1 km scale have sill values of 11.0 and 25.0 (per 100,000 people)^2^, respectively. Figure 3a–g show the scatter plots for downscaled OC mortality rates and the seven heavy metal concentrations in soil at the 1 km scale and the 95% confidence intervals for the Spearman’s rank correlation. The ranges of all of the 95% confidence intervals are around 0.08. Additionally, the 95% confidence intervals for Cd, Cr, and Pb had negative values; those for the remaining heavy metals exceeded 0. The strongest correlations (0.29 to 0.37) appeared in the scatter plot between the As concentration and OC mortality rate. In contrast, the weakest correlations (−0.12 to −0.04) appeared in the scatter plot between the Pb concentration and OC mortality rate.

### 3.2. Estimated Spatial Distribution of OC Mortality Rate and Local Moran Statistics

**Figure 4:**
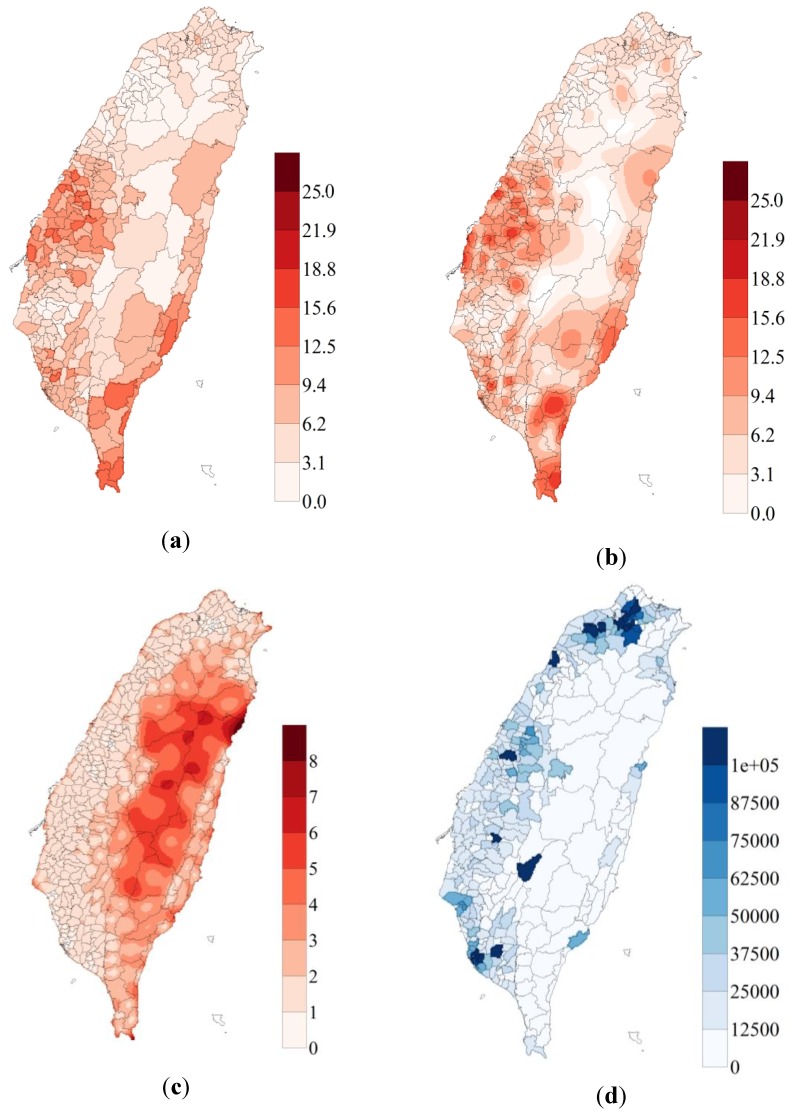
(**a**) Male oral cancer (OC) morality rate for districts (per 100,000 population); (**b**) downscaled Male OC mortality rate in a grid of 1 km × 1 km cells (per 100,000 population); (**c**) variance in OC rate predicted by area-to-point (ATP) Poisson kriging (per 100,000 population)^2^; (**d**) population in each district.

Figure 4b shows the OC mortality rate in a grid of 1 km × 1 km cells, which was estimated using ATP Poisson kriging on district-scale data (Figure 4a). High mortality rates were found in southern, eastern, and mid-western Taiwan. 

**Figure 5:**
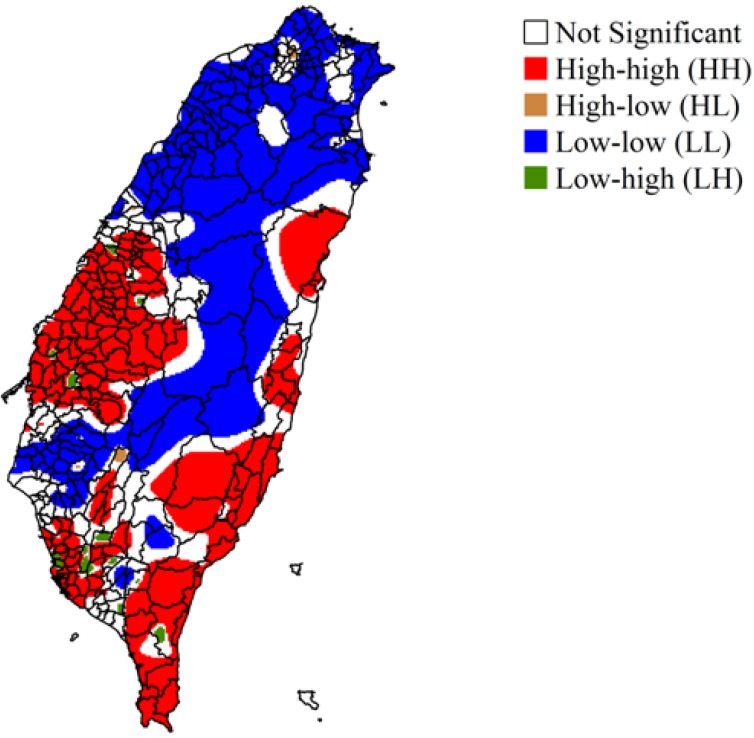
A local indicator of spatial association (LISA) map based on oral cancer mortality rate downscaled by area-to-point (ATP) Poisson kriging.

Figure 4c shows high variances in OC predictions obtained by ATP Poisson kriging for central Taiwan, including the central mountains. Figure 4d shows the population in each district. The populations in central Taiwan and the central Mountains are lower than those in all other districts. Figure 5 shows the LISA maps generated by a Local Moran statistical test with the OC rates downscaled by ATP Poisson kriging. Almost all hot spots throughout Taiwan yielded significant results in the Local Moran statistical test. The high OC mortality areas (HH) tended to be concentrated in southern, eastern, and mid-western areas of Taiwan. The low OC mortality areas (LL) tended to be concentrated in northern and central Taiwan.

### 3.3. Correlation between Land-use and OC Mortality Rate

**Figure 6:**
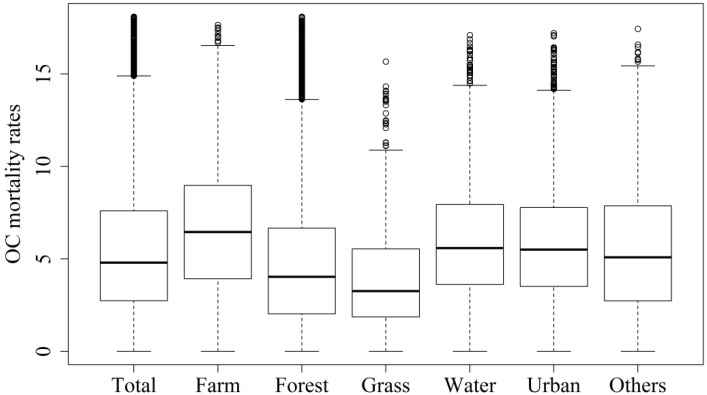
Boxplots of oral cancer (OC) mortality rates (per 100,000 people) downscaled by ATP Poisson kriging throughout study area (Total) and on land of different types—agricultural land (Farm), forestland (Forest), grassland (Grass), water body (Water), built-up (Urban), and others (Others).

For each land type, Figure 6 shows boxplots of OC mortality rates estimated by ATP Poisson kriging. The median OC mortality rate for agricultural land approximated 6.5 per hundred thousand people, which exceeded that for other land types. Grassland had the lowest mortality rate (3.0 per 100,000 population). The mean OC mortality rates for agriculture land, bodies of water, and built-up areas were significantly higher than the overall rate, but those in forestland and grassland were significantly lower. The variance in mortality rate was the lowest in built-up areas (9.8 (per 100,000 population)^2^) and highest in forestland (12.8 (per 100,000 population)^2^), where it exceeded the variance of mortality rate throughout the entire study area. The Kruskal-Wallis test also revealed a significant difference (*p*-value < 0.001) in OC mortality rates among land use types. The one-tailed multiple comparison test showed that agricultural land, water, and built-up land use types had significantly high OC mortality rates compared to other land use types.

Table 1 presents the proportions of hot spots on the six land types as estimated by ATP Poisson kriging. In each land type, the coverage by hot spots significantly differed from that in the overall study area. In agricultural land, bodies of water, built-up land, and land with other uses, coverage by hot spots was significantly larger than that of the overall study area. In contrast, coverage by hot spots was significantly lower in forestland and grassland compared to the overall study area. Agricultural land had the highest hotspot coverage (47%) while grassland had the lowest coverage (19%).

**Table 1:** Proportions of hot spots on land of six types, estimated using ATP Poisson kriging.

Land type	Hotspot (%)
**Farm**	46.4
**Forest**	25.8
**Grass**	19.3
**Water**	36.2
**Urban**	37.4
**Others**	41.4
**Entire area**	32.5

### 3.4. p-field Simulation of OC Mortality Rate

**Figure 7:**
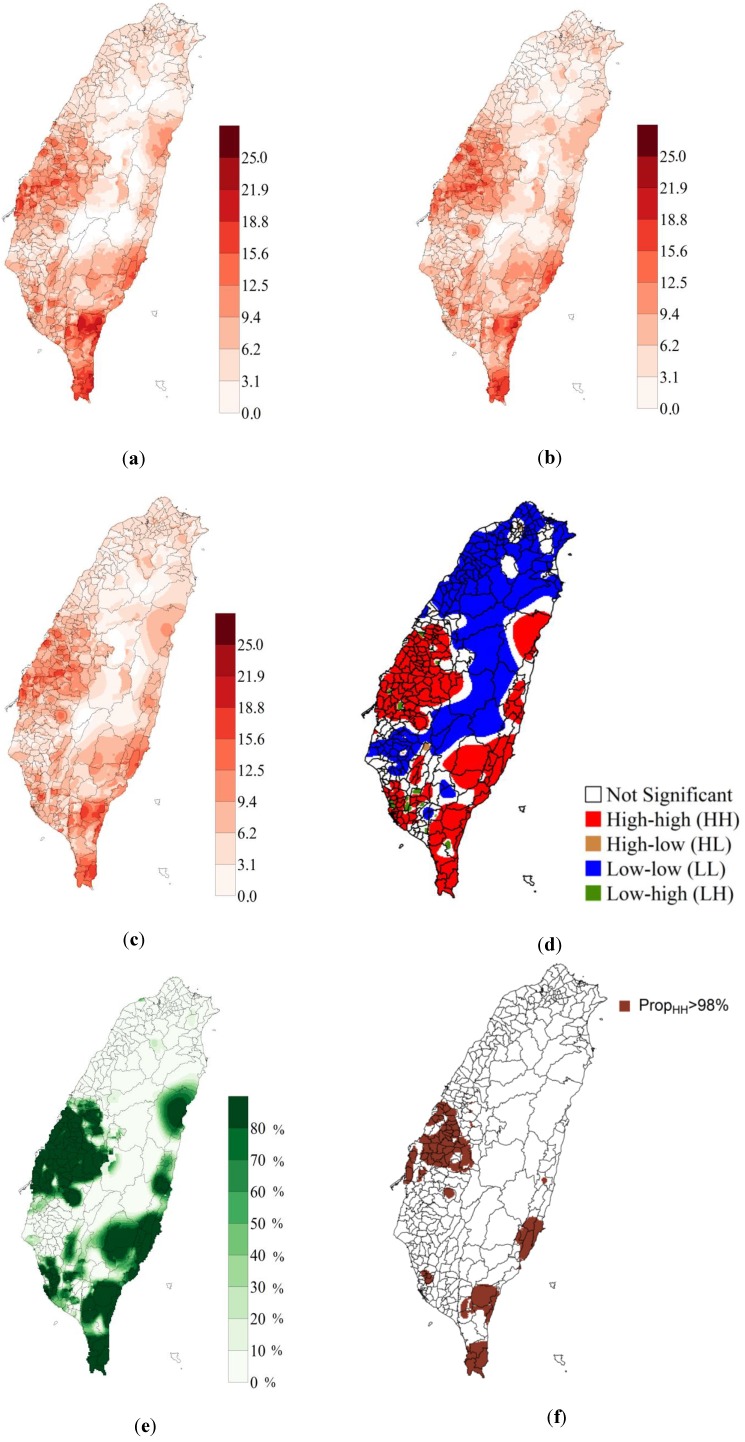
(**a**) The oral cancer (OC) mortality rate in the 1st realization (per 100,000 population); (**b**) OC mortality rate in the 8th realization (per 100,000 population); (**c**) mean OC morality rate in 1,000 p-field realizations (per 100,000 population); (**d**) LISA map based on mean OC mortality rate data in 1,000 p-field realizations; (**e**) proportion of study area covered by hot spots (High-High) in 1,000 p-field realizations; (**f**) area of simulated distribution of OC mortality rates over the entire study area with a high (98%) reliability (>0.6).

The spatial distribution of OC mortality rate was simulated 1,000 times using p-field simulation. Figure 7a–c show the OC mortality rate in the 1st and 8th of the 1,000 realizations, and the mean OC mortality rate of all 1,000 realizations, respectively. All three maps show that high mortality rates were concentrated in southern, eastern, and mid-western Taiwan. The uncertainty associated with the mortality maps was represented by the differences among the realizations. Figure 7d shows the LISA maps obtained by a Local Moran statistical test using the mean OC mortality rate from the 1,000 p-field simulations. Many areas in Taiwan yielded significant differences (hot spots and cold spots) in the Local Moran statistical tests. The hot spots (HH; High-High) of high OC mortality were in southern, eastern, and mid-western Taiwan (Figure 7d), and the cold spots (LL; Low-Low) of low OC mortality were in northern and central Taiwan. Figure 7e shows the proportion of hot spots (High-High) in 1,000 p-field realizations, which provides a measure of local uncertainty associated with a single location (grid) [20]. The areas with high (95%) hotspot coverage were located in southern, eastern, and mid-western Taiwan. The joint probabilities for the critical proportions 95%, 96%, 97%, 98%, and 99% (spatial uncertainties) of areas covered by hot spots were 0.22, 0.31, 0.42, 0.60, and 0.74, respectively. Figure 7f also shows the simulated distribution of OC mortality rates over the entire study area, which revealed a high (98%) reliability (>0.6). 

## 4. Discussion

This study downscaled Taiwan OC mortality rate data from the district scale to a 1 km × 1 km grid scale and then added soil sample data and land use data for identifying the relationship between the OC mortality rate and land use. The scatter plots and Spearman's rank correlation yielded the relationship between OC mortality and concentrations of the seven metals in the grid with 1 km cells. They show a weak correlation between the concentrations of the seven heavy metals in soil and the OC mortality rate at the 1 km scale. Local cluster analysis (LCA) identified hot spots of OC mortality (high-high; areas of high morality surrounded by areas with high mortality) in the 1 km grid cells that were clustered in southern, eastern, and mid-western Taiwan. High-high sites were significantly more prevalent on agricultural land and built-up land than on land with other uses. The 1,000 simulations of the OC mortality rate quantified local and spatial uncertainty in LCA results and further indicated OC mortality hotspots. 

### 4.1. Analysis of Variograms and the Relationship between OC and Heavy Metals

The variogram of male oral cancer mortality rates shows a spatial structure with a low nugget effect (Figure 2). Therefore, the mortality rate data show a high spatial autocorrelation. The large difference between the experimental variogram and the de-convoluted variogram was resulted from their large difference in scale. All scatter plots between the mortality rate and heavy metal concentrations (Figure 3a–g) reveal a very weak relationship. According to the interpretation of the Spearman's correlation coefficients [28], the correlations between OC mortality and the seven heavy metals are categorized to the low or weak level. Therefore, a weak correlation between the concentrations of heavy metals in soil and the rate of mortality associated with oral cancer at the 1 km scale was observed in the study area. The findings differ slightly from those of previous studies that determined that concentrations of As, Cr and Ni in soil are spatially correlated with OC mortality rate [2,3,4,11] at the district scale in Taiwan. We supposed that the 1 km scale is too small to reflect accurately the dose of heavy metal to which residents are exposed. Additionally, the deposition of heavy metals from airborne pollutants can be observed at a much smaller scale (near the emission source) than the scale used for soil sampling herein. Therefore, the highly spatial correlation between exposure to heavy metals and deposition of heavy metals from airborne pollutants is revealed at district level but not at the 1km scale.

### 4.2. Analysis of OC Mortality Patterns

Oral cancer is more concentrated in the mid-western and southern parts of Taiwan than elsewhere (Figure 4a). The distribution is similar to that of mortality rates, as estimated using ATP Poisson kriging (Figure 4b). The survival rate in each district is not determined. ATP Poisson kriging provides the variability of prevalence within each district (Figure 4c), and shows that districts in the central mountainous area exhibit high predicted variability because they have low populations.

**Figure 8:**
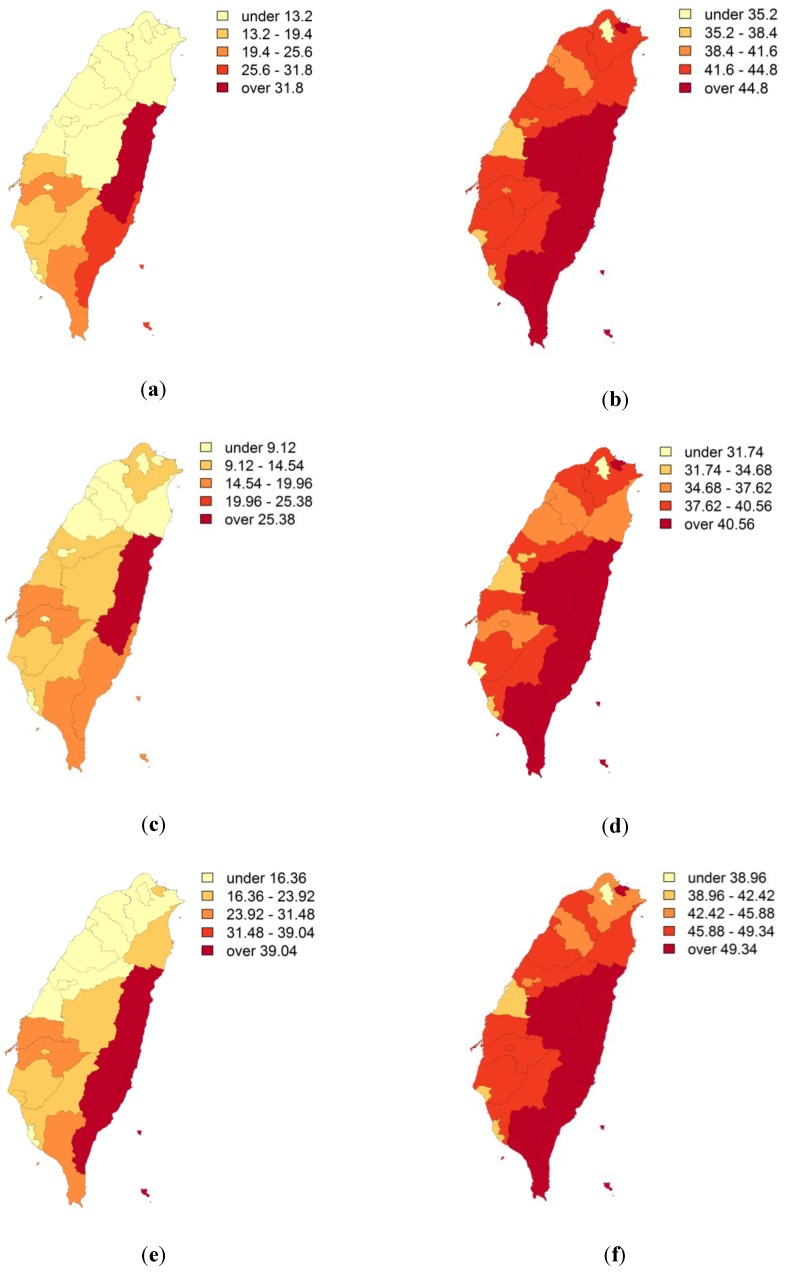
(**a**) Prevalence of betel quid chewing (%), (**b**) prevalence of cigarette smoking (%); (**c**) lower bound of 95% confidence interval of prevalence of betel quid chewing (%), and (**d**) prevalence of cigarette smoking (%); (**e**) upper bound of 95% confidence interval of prevalence of betel quid chewing (%), and (**f**) prevalence of cigarette smoking (%)

To identify the hot spots of OC mortality rate, LISA statistics were computed using data that were downscaled by ATP Poisson kriging. The kriging generates smoother OC rate surfaces. The smoothing effect of kriging tends to enhances spatial autocorrelation in the OC rate map, with the rate of inflating artificially cluster sizes [20]. The predicted variance in the OC rate explains the variation in the kriging estimates. Predicted variance was high only in the cluster in the center of the study area, which made that cluster unreliable. The unreliability in this area did not affect the results of further analysis. Hot spots associated with a high risk of OC mortality rate were concentrated in southern and eastern areas of Taiwan and in Changhua County in mid-western Taiwan. These areas have numerous electroplating plants, which can produce severe heavy metal pollution. Additionally, Changhua county ranks only 11th out of 23 counties in betel quid chewing in Taiwan [4,29,30,31]. The cigarette smoking and betel quid chewing rates that were obtained from a survey at the county level show that not all of the hot spots were in areas of high cigarette smoking or betel quid chewing rates (Figure 8a–f) [1]. Although the surveyed samples were not very large (46 to 1,381), they sufficed to confirm that cigarette smoking or betel quid chewing may not be the dominant factors in the accelerated incidence of OC in Changhua County. These findings support the association between OC mortality and the number of industrial facilities in a district [32,33]. 

However, the concentrations of the seven heavy metals in soil near many such facilities are not unusually high. We suspect that the deposition of heavy metals in soil from airborne pollutants may be observed at a much smaller scale (near the emission source) than that used herein in soil sampling. Based on a study conducted in Changhua County, Su *et al.* suggested that chewing betel quid may be just an inducer of oral cancer but that heavy metal pollutants act as promoters in the development of oral cancer [1]. Other studies have suggested that a high prevalence of betel quid chewing and cigarette smoking are the main causes of seriously high oral cancer mortality rates in southern and eastern Taiwan [1,4]. However, further surveys of betel quid chewing and cigarette smoking data with a high confidence level are needed. Additional survey data would improve analyses of associations between OC and cigarette smoking or betel quid chewing.

### 4.3. p-field Simulation of OC Mortality Rate

The distribution of mean OC mortality rate in 1,000 p-field realizations (Figure 7c) is similar to that downscaled by ATP Poisson kriging (Figure 4b). Based on this consistency, the distributions of the hot spots that are calculated from mean OC mortality rate data in 1,000 p-field realizations (Figure 7d) are expected to be similar to the distributions obtained after data are downscaled by ATP Poisson kriging (Figure 5). Additionally, the locations of the hot spots as determined from the OC mortality rate downscaled by ATP Poisson kriging are locations with high proportions of hot spots in the 1,000 realizations. However, in this study, the purpose of using p-field simulations to determine OC mortality rate was to quantify uncertainty in the LCA results. At a given critical proportion (95%, 96%, 97%, 98%, or 99%), a higher joint probability indicates greater reliability of mapped hotspot locations. The proportions of hot spots obtained over the entire study area in the 1,000 realizations are consistent with the hotspot distributions in each realization when the critical proportion of hot spots exceeded 98% with a joint probability >0.6 or exceeded 97.5% with a joint probability > 0.5. Kerry *et al.* [22] identified HH hotspot areas in which the proportion of hot spots exceeded 75%. Goovaerts [20] showed that a 90% proportion was reliable for identifying high mortality from cervical cancer whereas a 75% proportion yielded the least reliable results. Unlike Goovaerts [20] and Kerry *et al.* [22], this study calculated joint probabilities to evaluate spatial uncertainty in the reliability of delineated hot spots. Given the 98% proportion and high reliability (>0.6) obtained in this study, the area of the simulated distribution of OC mortality rates over the entire study area was significantly smaller than that determined by ATP Poisson kriging (Figure 7f). However, the OC mortality risk may be low in the mapping of HH areas when the critical proportion is greater than 98%. 

### 4.4. Spatial Patterns of OC Mortality in Different Land Use Types

The mean OC mortality rate and proportion of hot spots in agricultural land, near bodies of water, and in built-up areas significantly exceeded those in other land use types. This finding is consistent with the finding of Wei *et al.*, who found that mortality from cancer may be higher in areas with high proportions of agricultural land or in built-up areas than elsewhere [5]. In this study, land used for industrial plants was classified as built-up land. People who live near factories or in agricultural areas in Taiwan typically have a higher than normal risk of exposure to heavy metals because, in recent decades, wastewater discharged from factories into agricultural land has caused severe heavy metal contamination [3,29]. This finding is consistent with studies that human activity, most of which occurs in built-up areas and on agricultural land, is associated with increased concentrations of heavy metals in soil [6,7,8,9,10]. Therefore, soil can reveal the amounts of heavy metals in the environment and the risk of heavy metals exposure in nearby residents [1,34]. Many studies have shown that, at the district scale, heavy metal concentrations in soil are highly correlated with the development of OC [1,2,3,4,11,35]. This study found that exposure to heavy metals varies according to land use type and supports earlier studies showing that people who live near factories and in agricultural areas have a higher than normal risk of OC [1,11]. However, further research is needed to identify the main cause of the increased OC risk in people living in agricultural land and in urban areas in Taiwan.

## 5. Conclusions

Spatial correlations between the occurrence of OC and heavy metal concentrations in soil have been widely reported, however, this study revealed that these correlations were weak, especially given the spatial structure of OC occurrences. The de-convolution approach yielded variogram of OC mortality in a grid of 1km cells, and the scatter plots and Spearman's rank correlations yielded the relationship between the OC mortality and concentrations of the seven heavy metals in the grid with 1km cells. Land use showed a strong association with OC, particularly in agricultural land and built-up areas. However, mean OC mortality rates as well as the proportions of land covered by hot spots were significantly higher in agricultural land and in built-up areas than in other land use types. However, although this study identified a strong correlation between OC occurrence and land use, further evidence is needed to confirm this relationship and the high OC mortality rate in agricultural land and built-up areas. Stochastic simulation methods with Poisson kriging are highly reliable for delineating hot spots and for quantifying the local and spatial uncertainties in OC mortality rate and can provide the information that decision-makers and policy-makers need for effective environmental planning and management. The information obtained by the proposed approaches to calculating OC mortality can be considered when making land use decisions. 
